# Risk prediction models for non-suicidal self-injurious behavior in patient with depressive disorder: a protocol for systematic review and mata-analyisis

**DOI:** 10.1371/journal.pone.0321561

**Published:** 2025-04-17

**Authors:** Liu Huang, Xiao Liu, Jiao Xu, Ling Wang, Qiran Zhang

**Affiliations:** 1 College of Basic Medical Science, China Three Gorges University, Yichang, China; 2 School of Medicine, Xiangyang Polytechnic, Xiangyang, China; 3 Department of Integrated TCM and Western Medicine, Wuhan Mental Health Centre, Wuhan, China; 4 The First College of Clinical Medicine Science, China Three Gorges University, Yichang, China; Gachon University Gil Medical Center, REPUBLIC OF KOREA

## Abstract

**Introduction:**

Non-suicidal self-injury (NSSI) frequently occurs in patients with depressive disorder and often presents as burning or severe scratching. NSSI plays a crucial role in increasing the risk of self-injury in individuals with depressive disorder. Despite the progressive development of various risk prediction models to identify NSSI, there are significant differences in their overall predictive performance. This systematic review aims to evaluate the quality and applicability of these models in predicting NSSI among patients with depressive disorders.

**Methods and analysis:**

A systematic review with meta-analysis was conducted targeting patients with depressive disorder. We included studies on risk prediction models for NSSI behavior in this population that were developed and published. The primary outcome was NSSI behavior as reported by the prediction models. Predictive variables were measured at different disease stages in patients with depressive disorder, with no specific limitations on the prediction horizon. The intended use of the risk prediction model is to individualize the prediction of NSSI behavior of in patients with depressive disorder, thus facilitating the implementation of preventive measures to avoid adverse events. Databases, including China National Knowledge Infrastructure (CNKI), Wanfang Database, VIP Database, PubMed, Web of Science, Medline, and Embase, were searched from inception to March 2024 by two independently reviewers. Data extraction followed the guidelines outlined in the Checklist for Critical Appraisal and Data Extraction for Systematic Reviews of Prediction Modelling Studies (CHARMS). The risk of bias and applicability of the included studies were assessed using PROBAST. Descriptive statistical methods were employed to summarize the characteristics of the NSSI models and meta-analysis for model validation was conducted using Stata software.

**Conclusion:**

The study will systematically review the prediction models for NSSI in patients with depressive disorders to enhance clinical practice. This research will also assist clinicians in selecting effective prediction models for NSSI in this patient population.

## Introduction

Non-suicidal self-injury (NSSI) is defined by the intentional and direct infliction of harm to one's own body—such as self-cutting, burning, or severe scratching-without the intention of causing death and for purposes not recognized as socially acceptable [1]. Recently, NSSI has become increasingly prevalent worldwide, particularly among individuals with depressive disorders, thus emerging as a significant public health concern [2]. Research reveals that over 34.2% of those diagnosed with major depressive disorder have a history of NSSI, and individuals who engage in NSSI are at a heightened risk of receiving a diagnosis of major depressive disorder [3–4]. In addition, studies suggest that NSSI may significantly contribute to the risk of suicide within depressive disorders [5]. Notably, depressive disorders contribute substantially to the global disease burden, with a weighted lifetime prevalence of 3.4% in China [6].

NSSI emerges from a complex interplay of heterogeneous factors, and categorizing cases according to social characteristics could enhance the accuracy of early prediction.. With advancements in data-driven clinical decision-making, developing robust systems for the early identification, monitoring, and warning of NSSI has become increasingly crucial. Biological factors commonly associated with NSSI include atypicalbrain functions, neurochemical imbalances, dysfunctions of the HPA axis, altered pain perception, and epigenetic modifications [7]. Recently, various data models have been proposed to predict NSSI at various stages of depressive disorders [8–9]. Nonetheless, many of these models have been critiqued for their lack of

Several predictive models integrating multiple factors for anticipating NSSI have been developed. Wang et al. [9] identified childhood emotional abuse, along with sexual and physical abuse, as predictors of NSSI among depressed individuals, with childhood emotional abuse emerging as the most significant predictor.. Kim et al [8]. established a combination of factors to predict high-risk NSSI in patients with depression. However, to date, no study has systematically evaluated the quality and effectiveness of these predictive models.

In this study, we aim to systematically summarize the reported multivariable models developed for predicting NSSI in patient with depressive disorders, including their characteristics and performance. We will also evaluate whether these models have undergone external validation. Utilizing the Prediction Model Risk of Bias Assessment Tool (PROBAST), we will assess the risk of bias and applicability of the included studies that either develop or validate prediction models. For models with multiple validation studies, we will perform a meta-analysis to evaluate their performance and calibration, thereby providing more precise effect estimates.

## Method and analysis

We will design and conduct this systematic review in accordance with the guidelines outlined by the Preferred Reporting Items for Systematic Review and Meta-Analyses (PRISMA) Protocol [10]. The Checklist for Critical Appraisal and Data Extraction of Systematic Reviews of Prediction Modelling Studies (CHARMS) was utilized for the assessment and extraction of data [11]. This framework supports the formulation of review's objectives, development of the search strategy, and establishment of criteria for study inclusion and exclusion [12]. The study protocol has been registered with PROSPERO under registration number of CRD42024524274.

### Literature search

**Fig 1:**
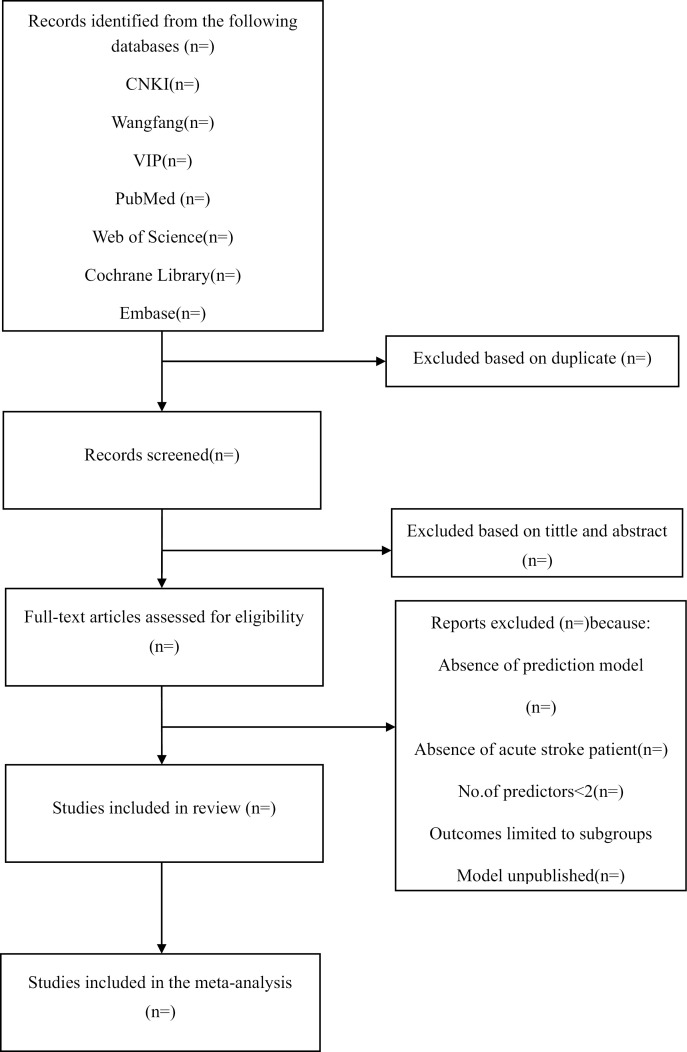
Flowchart of the literature search and section.

We systematically searched both Chinese and English databases, including China National Knowledge Infrastructure (CNKI), Wangfang Database, China Science and Technology Journal Database (VIP), PubMed, Embase, The Cochrane and Web of science, from their inception through March 2024. The search strategy in supporting information 2. Two independent investigators will perform the literature search and screening, with any discrepancies resolved by a senior author. Additionally, we will seek out other relevant studies, including unpublished and ongoing articles. The retrieval flowchart is shown in Fig 1.

### Eligibility criteria

We will include all cohort studies that described the development and validation of original multivariable models for predicting NSSI behavior in patients with depressive disorders. Articles will be screened and selected based on both inclusion and exclusion criteria. Inclusion criteria for studies are as follows: (1) patients with depressive disorders;(2) Observational study design;(3) Reporting of a prediction model with internal validation; (4) Outcome of interest being NNSI behavior. The exclusion criteria are: (1) studies that did not develop a prediction model; (2) Unavailability of full text; (3) Fewer than two predictors. For the systematic review, we used the PICOTS framework (Population, Intervention, Comparator, Outcomes, Timing, Setting), as outlined in Table 1. According to PICOTS guidelines, our study design does not include randomized controlled trials; therefore competing models were not considered as comparators.

**Table 1:** The item of formulating the review’s aim, search strategy, study inclusion and exclusion criteria with PICOTS guidelines.

Item	Definite
P (Population)	Patients with depressive disorder
I (Intervention)	Risk prediction models for NSSI behavior in patient with depressive disorder that were developed and published (predictors ≥ 2).
C (Comparator)	No competing model
O (Outcome)	non-suicidal self-injurious reported by prediction models
T (Timing)	Predictive variables measured at different disease stages in patient with depressive disorder; no specific limitation applied in prediction horizon
Setting	The intended use of the risk prediction model is to individualize the prediction of NSSI behavior of in patients with depressive disorder, facilitating the implementation of preventive measures to prevent adverse events.

### Data abstraction

Two researchers independently extracted data following the recommendations of the CHARMS checklist and verified their findings. In cases of uncertainty regarding data extraction, the two researchers will consult and discuss the issues until an agreement is reached and proceed with data extraction. If disagreements persist, neutral third-party reviewer will be engaged to resolve the issues. The third reviewer will extract data based on CHARMS checklists. Finally, team will reach a consensus through discussion.

The extracted information will be categorized into two sections: general information and model information. General information will include details such as author, year, research design, model name, assessment tool, publication, and country. Model information will cover candidate variables, sample size, modeling methods, handling of missing data, predictive factor assessment, discriminative power, and model evaluation.

This study independently assessed the quality of included literature using PROBAST, a tool developed by Wolff et al. (2019) to evaluate the risk of bias and applicability of the literature.[13]. PROBAST is organized into four domains: participants, predictors, outcome, and analysis, comprising a total of 20 signaling questions designed to facilitate a structured judgment of risk of bias (ROB). ROB arises from deficiencies in study design, conduct, or analysis, leading to. systematically distorted estimates of model predictive performance. Each question was answered on a scale of yes, probably yes, no, probably no, no information. Domains were classified as low, high and unclear risk of bias. Details for the assessment rules are summarized in Table 2.

**Table 2:** Summary of assessment of risk of bias and concerns regarding applicability.

1.participants	2.predictor	3.Outcome	4.Analysis
1.1 Were appropriate data sources used, for example, cohort, randomized controlled trial (RCT) or nested case–control study data?	2.1 Were predictors defined and assessed in a similar way for all participants?	3.1 Was the outcome determined appropriately?	4.1 Were there a reasonable number of participants with the outcome?
1.2 Were all inclusions and exclusions of participants appropriate?	2.2 Were predictor assessments made without knowledge of outcome data?	3.2 Was a prespecified or standard outcome definition used?	4.2 Were continuous and categorical predictors handled appropriately?
	2.3 Are all predictors available at the time the model is intended to be used?	3.3 Were predictors excluded from the outcome definition?	4.3 Were all enrolled participants included in the analysis?
		3.4 Was the outcome defined and determined in a similar way for all participants?	4.4 Were participants with missing data handled appropriately?
		3.5 Was the outcome determined without knowledge of predictor information?	4.5 Was selection of predictors based on univariable analysis avoided?
		3.6 Was the time interval between predictor assessment and outcome determination appropriate?	4.6 Were complexities in the data (e.g., censoring, competing risks and sampling of control participants) accounted for appropriately?
			4.7 Were relevant model performance measures evaluated appropriately?
			4.8 Were model overfitting and optimism in model performance ac counted for?
			4.9 Do predictors and their assigned weights in the final model correspond to the results from the reported multivariable analysis?

### Statistical analysis

We employ descriptive statistical methods to summarize the characteristics of NSSI models. For categorical variables, we will calculate frequencies or percentages, while for continuous variables, we will compute means, medians, and interquartile ranges (IQRs). Meta-analysis of the validated models was performed using Stata software. Heterogeneity was assessed using the I^2^ index and Cochrane Q test. The criteria for heterogeneity are evaluated using this index, which mainly categorized into low, medium, and high [14]. In the presence of significant heterogeneity, a random effects model will be applied to merge effect sizes and address heterogeneity. Sources of heterogeneity will be explored through subgroup analysis and meta-regression. Additionally, sensitivity analysis will be conducted to exclude outlier studies, and meta-analysis will be repeated to compare results with those obtained without excluding anomalies. We will also perform a meta-analysis of C-statistics for prediction models.

#### Sensitivity analysis.

We can also perform a sensitivity analysis to exploit the reliability of the research findings and ascertain the extent to which they are affected by other influencing factors..

#### Subgroup analysis.

We will assess the heterogeneity of meta-analysis to determine whether subgroup analysis is necessary

#### Ethics and dissemination.

Ethical approval was deemed unnecessary, as all data will be sourced from previously published studies. We intend to publish these findings in a peer-reviewed journal. The PROSPERO registration number for this study is CRD42024524274.

## Result

### Patient and public involvement

This study constitutes a systematic review of the existing literature and was conducted without direct involvement from patients or the public in its design, execution, or reporting.

### Amendments

The protocol for this systematic review will be amended as needed during the peer review process. The author would publish when the study is complete.

## Discussion

To date, numerous prediction models have been developed to identify the NSSI, but many may not have been validated due to various limitations. We will systematically review the published studies on these prediction models for NSSI to enhance clinical practice. The findings aim to assist clinicians in selecting effective NSSI prediction models for patients with depressive disorders. We evaluate both the internal and external validity of these prediction model using various indicators. The primary limitation of this study is that it is a systematic review of existing literature on NSSI prediction models for depression patients and did not generate any new data. In the future, it may provide a theoretical foundation for the development of prediction or alert systems for NSSI and enable earlier targeted preventive measures.

## Supporting information

S1 File Search strategy for the each English databases. (DOCX)

S2 File PRISMA-P 2015 Checklist. (DOCX)
