# Supplementary material for: Risk prediction models for non-suicidal self-injurious behavior in patient with depressive disorder: a protocol for systematic review and mata-analyisis
Source: PLoS One. 2025 Apr 17;20(4):e0321561. doi: 10.1371/journal.pone.0321561 (PMC12005486; doi:10.1371/journal.pone.0321561)
Supplement: S1 File — (DOCX) [file pone.0321561.s001.docx]

**Table 1. Search strategy for the each English databases.**

| **Databases** | **Search strategy** |
| --- | --- |
| **Pubmed** | #1:(Depressive Disoder [MeSH]) OR (depressive Neuros* [Title/Abstract]) OR (Endogenous Depression [Title/Abstract]) OR (Melancholia [Title/Abstract]) OR (Unipolar Depression [Title/Abstract]) OR (Depressive Syndrome [Title/Abstract]) OR (Neurotic Depression [Title/Abstract]  #2: (Self-injurious behavior [MeSH Terms]) OR (non-suicidal self-injurious behavior [Title/Abstract]) OR (NSSI[Title/Abstract]) OR (non-suicidal selt-harm [Title/Abstract]) OR (nonsuicidal self-injury [Title/Abstract])  #3: (Predict* [Title/Abstract]) OR (progn* [Title/Abstract]) OR (risk predict* [Title/Abstract]) OR (risk score [Title/Abstract]) OR (risk calculation [Title/Abstract]) OR (risk assessment [Title/Abstract]) OR (predictive factor [Title/Abstract]) OR (prediction model [Title/Abstract])  #4: #1 AND #2 AND #3 |
| **Web of science** | **#1:** **(((((**TS=(“Depressive Disoder”) OR (TS=（“depressive Neuros* ”) OR (TS=（“Endogenous Depression”) OR (TS=（“Melancholia ”) OR (TS=（“Unipolar Depression ”) OR (TS=（“Depressive Syndrome ”) OR (TS=“Neurotic Depression ”)  **#2：((((**TS**=**(“Self-injurious behavior ”) OR (TS=（“non-suicidal self-injurious behavior ”) OR（TS= (“NSSI”) OR （TS=(“non-suicidal selt-harm”) OR (TS=“nonsuicidal self-injury ”)  **#3：**((((((((TS=(“Predict* ”) OR TS=(“progn* ”) OR TS= (“risk predict* ”) OR TS= (“risk score ”) OR TS =(“risk calculation ”) OR TS= (“risk assessment” ) OR TS= (“predictive factor ”) OR TS=(“prediction model ”)  #4: #1 AND #2 AND #3 |
| **Embase** | **#1:** ’Depressive Disoder’：ti, ab, kw OR ’depressive Neuros*’: ti, ab, kw OR’Endogenous Depression’: ti, ab, kw OR ’Melancholia’: ti, ab, kw OR ’Unipolar Depression’: ti, ab, kw OR ’Depressive Syndrome’: ti, ab, kw OR ’Neurotic Depression’: ti, ab, kw  **#2:** ’Self-injurious behavior ’：ti , ab, kw OR ’non-suicidal self-injurious behavior ’ ：ti, ab, kw OR’NSSI’ ：ti, ab, kw OR ’non-suicidal selt-harm’ ：ti, ab, kw OR ’nonsuicidal self-injury ’ ：ti, ab, kw  **#3：** Predict*’：ti, ab, kw OR ’progn*’ ：ti, ab, kw OR risk predict* ’ ：ti, ab, kw” OR risk score ’ ：ti, ab, kw OR TS =(“risk calculation ’ ：ti, ab, kw OR TS= (“risk assessment’ ：ti, ab, kw OR predictive factor ’ ：ti, ab, kw OR prediction model ’ ：ti, ab, kw  #4: #1 AND #2 AND #3 |
| **Medline** | #1:AB“Depressive Disoder” OR AB“depressive Neuros* ” OR AB“Endogenous Depression”OR AB “Melancholia ”OR AB“Unipolar Depression ”OR AB“Depressive Syndrome ” OR AB“Neurotic Depression ”  #2：AB“Self-injurious behavior ” OR AB“non-suicidal self-injurious behavior ”OR AB“NSSI” OR AB“non-suicidal selt-harm” OR AB“nonsuicidal self-injury ”  #3：AB “Predict* ” OR AB “progn* ”OR AB“risk predict* ” OR AB“risk score ”OR AB“risk calculation ” OR AB“risk assessment” OR AB“predictive factor ”OR AB“prediction model ”  #4: #1 AND #2 AND #3 |
| CNKI | #1:SU=“抑郁”OR “抑郁症” OR “抑郁障碍”  #2：SU=杀性自伤” OR“非自杀性自伤行为” + NSSI  #3:SU=模型+预测模型+因素+风险因素+危险因素  #1 AND #2 AND #3 |
| Wangfang | #1:主题=“抑郁”OR “抑郁症” OR “抑郁障碍”  #2：主题=杀性自伤” OR“非自杀性自伤行为” + NSSI  #3:主题=模型+预测模型+因素+风险因素+危险因素  #1 AND #2 AND #3 |
| VIP | #1:K=“抑郁”OR “抑郁症” OR “抑郁障碍”  #2：K=杀性自伤” OR“非自杀性自伤行为” + NSSI  #3:SU=模型+预测模型+因素+风险因素+危险因素  #1 AND #2 AND #3 |
